# Parasite Burden of *Trypanosoma cruzi* in Whole Blood and Buffy Coat Determined by Real-Time PCR in Individuals with Chronic Chagas Disease

**DOI:** 10.3390/microorganisms12020249

**Published:** 2024-01-25

**Authors:** Daniela Liempi, Inés Zulantay, Nelson M. Varela, Mauricio Canals, Andrés Guevara, Nicolás Poulsen, Werner Apt

**Affiliations:** 1 Institute of Parasitology, Faculty of Medicine, Austral University of Chile, Valdivia 5090000, Chile; 2 Master’s Program in Parasitology, Graduate School, Faculty of Medicine, University of Chile, Santiago 8380453, Chile; 3 Basic-Clinical Parasitology Laboratory, Cellular and Molecular Biology Program, Institute of Biomedical Sciences, Faculty of Medicine, University of Chile, Santiago 8380453, Chile; wapt@uchile.cl; 4 Basic-Clinical Oncology Department, Faculty of Medicine, University of Chile, Santiago 8500000, Chile; nvarela@uchile.cl; 5 Department of Eastern Medicine and Environmental Health Program, School of Public Health, Faculty of Medicine, University of Chile, Santiago 8380453, Chile; mcanals@uchile.cl; 6 School of Medical Technology, Faculty of Medicine, University of Chile, Santiago 8380000, Chile; andresguevara@ug.uchile.cl (A.G.); nicolaspoulsen@ug.uchile.cl (N.P.)

**Keywords:** chronic Chagas disease, buffy coat, guanidine, parasitemia, real-time PCR

## Abstract

The objective of this study was to compare, by qPCR, the circulating blood parasite load of *Trypanosoma cruzi* in the buffy coat, and in whole blood mixed with boiled and unboiled guanidine hydrochloride-EDTA buffer, of individuals with chronic ChD. The concentration and purity of DNA were evaluated in a Nanodrop Denovix DS-11FX Series Spectrophotometer (DeNovix Inc., Wilmington, NC, USA). The parasite load was determined with the Taqman^®^ qPCR system using a Stratagene Mx3000P thermocycler (Agilent Technologies, Santa Clara, CA, USA) with Cruzi 1 and Cruzi 2 satellite primers. Student’s *t*-test with Bonferroni correction, Chi-squared (χ^2^) tests and Spearman’s correlation coefficient were applied. The concentration and purity of DNA were higher in the buffy coat. Parasite DNA was detected and quantifiable in the three types of samples in seven patients, without statistically significant differences in the parasite load obtained. Higher correlations were found between the total DNA concentrations and the parasite loads obtained in the samples of the buffy coat.

## 1. Introduction

Chagas disease (ChD) is a parasitic disease caused by the hemoflagellate protozoan *Trypanosoma cruzi*, which infects mammals and triatomines. It can be acquired or congenital, affecting various organs and systems to a variable degree, especially the heart and digestive tract [1].

This anthropozoonosis, endemic in 21 Latin American countries, is linked to cultural and socioeconomic factors, being considered among the most neglected tropical diseases in Latin America [2,3,4] due to research and development gaps related to diagnosis and treatment [5]. The World Health Organization (WHO) estimates that between six and seven million people are infected worldwide, mainly in Latin America [4], causing more than 10,000 deaths per year [6,7]. The endemic area in Chile is located between the Arica-Parinacota (18°30′ S) and O’Higgins (34°36′ S) regions, with 873,415 people at risk, mainly in rural and peri-urban areas [6,8]. This parasitosis has become a global public health problem in recent decades, due to the migration of infected people from rural to urban areas and migratory flows that have spread the disease to other continents [9].

ChD occurs in two stages: acute and chronic [9]. Many parasites circulate in the blood during the acute phase; the symptoms are variable, and, in most cases, they are absent or mild and nonspecific, resolving spontaneously after a few weeks [10]. Subsequently, the chronic phase ensues, initially with an indeterminate or latent form. Approximately 70% of people are asymptomatic, while 20–30% develop cardiac abnormalities (cardiomyopathies, arrhythmias), and 10% develop digestive disorders (megavisceras) several years or decades later [10,11,12].

Diagnostic methods differ depending on the stage of infection, favoring direct methods in the acute phase and serological methods in the chronic phase [13]. Molecular methods detect the parasite in all phases of the disease, and due to their higher sensitivity, provide positive results in infected patients who are negative in traditional parasitological tests [14]. It has been shown that PCR has greater sensitivity than other diagnostic techniques to blood samples from infected individuals, both in the acute and chronic stages [15,16]. qPCR procedures have been standardized for the detection of *T. cruzi* in blood samples preserved in 6M guanidine-HCl/0.2M EDTA buffer (GEB), in addition to performing analytical validation and clinical evaluation of multiplex qPCR based on TaqMan^®^ probes to detect and quantify satellite DNA (satDNA) or kinetoplast DNA (kDNA) of *T. cruzi* [17,18]. Isolation of the parasite from the bloodstream in the chronic phase remains a challenge. There are different strategies and protocols for using qPCR to determine the parasite load in the blood of individuals with ChD, which vary in the volume and fractionation of the sample, use of anticoagulants and/or preservatives, storage conditions and incubation, among other variables [17,19,20], which could influence both the purity and concentration of the DNA extracted from biological samples, as well as the estimation of the parasite load of *T. cruzi.*

The present study considered it relevant to study the levels of DNA concentration present in the peripheral blood samples of patients with chronic ChD, using different methodologies for obtaining and processing samples, as well as for studying their quality and purity, to establish if these variants influence the detection of the parasite load. Therefore, the purpose of this study was the comparison of the circulating parasite load of *T. cruzi* using qPCR DNA extracted from whole blood samples mixed with boiled and nonboiled GEB, and in the buffy coat (BC), obtained from individuals with chronic ChD.

## 2. Materials and Methods

### 2.1. Ethics Statement

The individuals selected for this study signed an informed consent form previously approved by the Ethics Committee for Research in Human Beings (CEISH) of the Faculty of Medicine of the University of Chile (Resolution N° 011/2017).

### 2.2. Study Population

Fifty-three individuals with a history of untreated chronic ChD, coming from different rural sectors of the Combarbalá commune, Coquimbo Region, were studied using ELISA and IFI IgG assays for infection by *T. cruzi* [8,21]. The samples that were positive or indeterminate were sent for confirmation to the Public Health Institute of Chile, Parasitology Section, as established by the current regulations of the Ministry of Health of Chile [8,22].

### 2.3. Obtaining and Processing of Biological Samples

Ten mL of peripheral blood was obtained from each patient by venous puncture and divided among three tubes: 4 mL in a tube without additives (to obtain serum for serological tests), 3 mL in a tube with EDTA anticoagulant (to obtain the BC and perform molecular tests) and 3 mL in a tube with GEB in a 1:1 ratio (for molecular tests). The BC was separated from the samples obtained in EDTA by centrifuging at 1500× *g* for 15 min, less than 72 h after its collection. These were preserved at −80 °C until DNA extraction (Omega Bio-tek, Inc., Norcross, Giorgia, USA). Once the serological tests were carried out, the whole blood obtained in the GEB was separated into two aliquots of 2 mL each; one aliquot was boiled at 98 °C for 15 min [17,19,23], while the other was kept without boiling, storing both at 4 °C until DNA extraction [19,24].

### 2.4. DNA Extraction

A 250 µL volume was extracted from all samples, using the E.Z.N.A.^®^ Blood DNA Mini Kit (Omega Bio-Tek, USA) according to the manufacturer’s instructions. The DNA was stored at −20 °C until concentration and purity were determined by spectrophotometry.

### 2.5. Determination of DNA Concentration and Purity

The concentration and purity of the DNA of the three types of samples was determined using a NanoDrop Denovix DS-11FX Series Spectrophotometer (DeNovix Inc., Wilmington, DE, USA) to measure DNA absorbances at 260/280 nm and 260/230 nm [25,26], following the manufacturer’s recommendations. The classification of purity ranges according to wavelength were based on those established by the Quality Control Department of the National DNA Bank, Carlos III [27,28,29].

### 2.6. Trypanosoma cruzi Strains and Development of the Standard Curve

For the quantification of *T. cruzi* DNA in clinical samples, a standard curve was prepared from the extraction of DNA from epimastigotes of clones belonging to strains Dm28c (TcI) and Y (TcII), which are DTUs circulating with high frequency in Chile [30,31], from axenic cultures. A 1:1 mixture of the parasite strains was made containing 1 × 10^6^ epimastigotes, considering that each parasite cell has approximately 200 fg of DNA; that is, 1,000,000 parasites-eq/mL corresponds to a concentration of 0.2 ng/μL of DNA [32,33,34,35]. Corresponding volumes of DNA were taken from each strain and diluted in the elution buffer of the extraction kit, obtaining solutions with a concentration of 0.4 ng/μL DNA. To confirm the required value, the concentration of the mixture was quantified in a Qubit^®^ ds DNA HS Assay Kit spectrophotometer in a Qubit^®^ 3 fluorimeter (Thermo Fischer, Scientific, Loughborough, UK). Subsequently, the mixture was diluted to reach a concentration of 0.2 ng/μL of DNA, obtaining the mother tube from which the different points of the calibration curve were prepared. From the tube with 1 × 10^6^ parasites, ten-fold serial dilutions (100,000, 10,000, 1000, 100, 10, 1 and 0.1 par-eq/mL) were prepared, using a pool of negative DNA (from healthy individuals without ChD) [17,31] with the three types of samples (boiled GEB, nonboiled GEB and BC). A standard curve was constructed for each methodology used. Each point on the curve was evaluated in duplicate in each qPCR assay. Two quantitative controls (0.1 and 10 par-eq/mL) were incorporated in each of the assays, which were considered as test samples. To evaluate the performance of the standard curve and validate the analytical run, it was verified that the parameters of reaction efficiency (Eff: 90–110%), linearity (R^2^: >0.98) and slope (Y: −3.1 to −3.6) met the pre-established acceptance criteria [36].

### 2.7. Quantification of T. cruzi Using qPCR-TaqMan^®^ System

The parasite load in the study samples was quantified by qPCR assays with the detection system with TaqMan^®^ probes, in a Stratagene Mx3000P thermocycler (Agilent Technologies, Santa Clara, CA, USA). The primers used in this study are described in Table 1.

The final reaction volume was 20 µL. The preincubation was performed for 1 cycle at temperatures of 25 °C for 1 s and 95 °C for 10 min, followed by 40 cycles at 95 °C for 15 s and 58 °C for 1 min. The target sequence in this reaction (satDNA) and previously optimized exogenous internal positive control (Exo-IPC) TaqMan^®^ reagents (Applied Biosystems, Foster City, CA, USA) were coamplified. As a negative control we used nuclease-free water and a pool of human DNA from peripheral blood from individuals with negative serology for ChD in the three types of samples. The reference DTUs TcI (Dm28c) and TcII (Y) were used as positive controls. Each assay was performed in duplicate: standard curve, negative controls, positive controls and *T. cruzi* parasite load controls incorporated as test samples, corresponding to the lowest and highest points of the curve. qPCR has a detection limit of 0.1 and 0.01 par-eq/mL [34]. However, parasitemia less than 0.1 par-eq/mL, that is, below the last point of the standard curve incorporated in the study, in our opinion is a nonquantifiable positive result, so lower values would correspond to DNA traces [37].

### 2.8. Statistical Analysis

This is an experimental comparative study. Concentrations (ng/µL) and parasite loads (par-eq/mL) were considered as quantitative variables, while the qualitative variables were the results of DNA purity, established in ranges. Student’s *t*-test for dependent samples was used with Bonferroni correction (α = 0.016) to compare the DNA concentrations and parasite loads of each of the sample types. Sequential Chi-squared (χ^2^) tests were used to compare the DNA purity of the three sample types and the number of samples with detectable DNA in each of the groups. Spearman correlation (rs) was used to establish the association between DNA concentration and parasite load for nonparametric variables; a significance level of α = 0.05 was used.

## 3. Results

**Figure 1:**
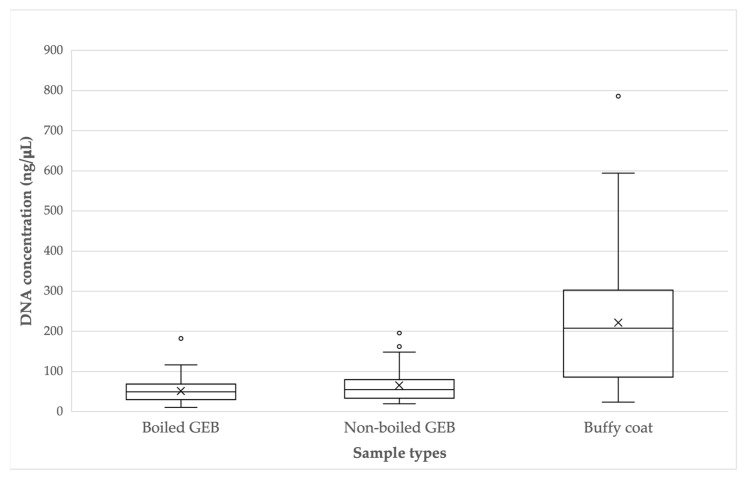
DNA concentration (ng/μL) according to sample types.

The DNA concentrations obtained in the samples in boiled GEB fluctuated between 10.758 ng/µL and 182.530 ng/µL (mean: 59.5, median: 49.3, standard deviation: ±30.5), while the concentrations of DNA from the nonboiled GEB samples ranged from 19.534 ng/µL to 195.868 ng/µL (mean: 65.6, median: 54.8, standard deviation: ±42.0). The DNA concentrations in buffy coat samples were between 24.11 ng/µL and 786.07 ng/µL (mean: 221.8, median: 207.7, standard deviation: ±160.4). Outliers are observed in each group of samples (Figure 1). The *t*-tests established that there was a higher concentration of DNA in the samples obtained from the BC, significantly greater than the samples from boiled GEB (t = −7.51, *p* < 0.001) and nonboiled GEB (t = −6.59, *p* < 0.01). There was no significant difference between the concentrations of the boiled GEB and nonboiled GEB samples (t = −2.06, *p* = 0.044).

There were significant differences in the purity of the DNA between the types of samples analyzed (χ^2^ = 21.03, *p* = 0.0003); the buffy coat samples had purer DNA than the other types of samples, supported by the fact that they did not present evidence of contamination. When comparing the samples of boiled GEB and nonboiled GEB, it was established that there are differences (*p* = 0.022), considering that the samples in nonboiled GEB present a lower number of contaminated samples and add up to a greater number of samples within the optimal and acceptable ranges of purity (Table 2).

**Figure 2:**
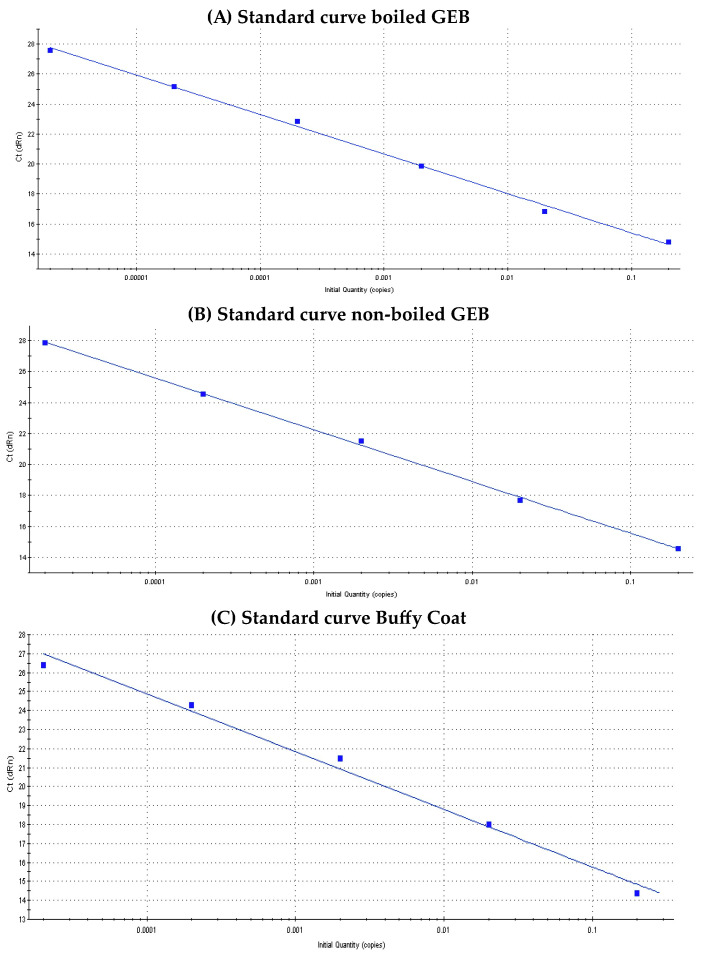
Standard curves of *Trypanosoma cruzi*, according to sample type.

Figure 2 shows the standard qPCR curves of *T. cruzi* according to sample type. The regression coefficient (R^2^), slope and efficiency (%) values for each of them were: (a) Standard curve boiled GEB: R^2^ = 0.997, slope = −2.628, Eff = 140.2% (b) Standard curve non-boiled GEB: R^2^ = 0.999, slope = −3.345, Eff = 99% and (c) Standard curve BC: R^2^ = 0.989, slope = −3.03, Eff: 113.8%.

Amplification of the IPC control (positive results) was observed in 100% of the samples, whose Ct (cycle threshold) allowed validating and determining that in the qPCR reaction there was no inhibition or false negatives due to the absence of DNA.

*T. cruzi* DNA was detected (in at least one of the three types of samples analyzed) in 26 patients (49.1%), while in 27 cases (50.9%) no parasite DNA was detected in any of the sample types. The presence of *T. cruzi* DNA was determined in 19 samples from boiled GEB (35.8%), 17 from nonboiled GEB (32%), and 18 from BC (34%). There were no significant differences between the number of positive samples according to sample type (*χ*^2^ = 0.168, *p* = 0.92). Parasite DNA was detected in all three types of samples in 10 patients (18.8%). Eight positive cases were detected in two types of samples (boiled GEB and nonboiled GEB, boiled GEB and BC, or nonboiled GEB and BC), the same as if only one type of sample had been analyzed (boiled GEB, nonboiled GEB or BC) (Table 3).

The results of the quantification of parasite load (par-eq/mL) of *T. cruzi* determined by qPCR-Taqman^®^ in the different types of samples are shown in Table 4.

The parasitemia varied from 0.04 to 1.29 par-eq/mL (mean: 0.27 par-eq/mL, SD: ±0.37) in boiled GEB, 0.37 to 7.55 par-eq/mL (mean: 2.11 par-eq/mL, SD: ±1.96) in nonboiled GEB, and 0.03 to 18.43 par-eq/mL (mean: 2.9 par-eq/mL, SD: ±5.1) in BC. The ranges of detectable parasitemia according to the type of sample are shown in Table 5.

Considering the quantification limit of parasitemia established in our laboratory (≥0.1 par-eq/mL), reliable quantification of parasitemia was determined in nine cases in boiled GEB, 17 cases in nonboiled GEB and 14 cases in BC. There were no significant differences in the number of quantifiable (>0.1 par-eq/mL) samples according to the type of sample (*χ*^2^ = 3.27, *p* = 0.19).

**Figure 3:**
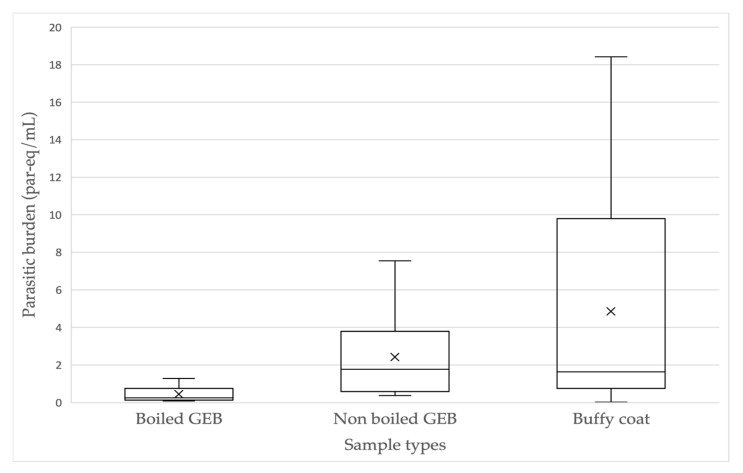
Parasite burden (par-eq/mL) of *Trypanosoma cruzi*, determined simultaneously in three typeof samples in 10 patients with chronic ChD.

As shown above, in 10 patients *T. cruzi* was detected in all sample types (Table 3). The parasite burden ranges were 0.08 to 1.29 par-eq/mL (mean: 0.45 par-eq/mL, SD: ±0.44) for boiled GEB, 0.37 to 7.55 par-eq/mL (mean: 2.42 par-eq/mL, SD: ±2.31) for nonboiled GEB and 0.03 to 18.43 par-eq/mL (mean: 4.85 par-eq/mL, SD: ±6.26) for BC (Figure 3). A statistically significant difference was only found between the burden in boiled GEB and nonboiled GEB (t = −3.069, *p* = 0.013), with higher burden in nonboiled GEB samples. A difference was found between the burden obtained in boiled GEB and BC (t = −2.33, *p* = 0.044), but not between nonboiled GEB and BC (t = −1.25, *p* = 0.24).

However, considering the quantifiable positive results (≥0.1 par-eq/mL), the parasitemia value could only be obtained in the three types of samples in seven patients. There was a significant difference between the parasite burden estimated in boiled GEB versus nonboiled GEB (*p* = 0.016), but not between boiled GEB versus BC (*p* = 0.06) or nonboiled GEB versus BC (*p* = 0.303).

No correlation (Spearman) could be demonstrated between the DNA concentration and the parasite burden obtained in samples of boiled GEB (rs = −0.03), the samples in nonboiled GEB (rs = −0.23) or those from BC (rs = 0.2). However, analyzing only the cases where a quantifiable parasite burden was obtained (≥0.1 par-eq/mL), the rs were 0.46 (boiled GEB), 0.57 (nonboiled GEB) and 0.96 (BC), which indicate a higher correlation, showing that as the concentration of DNA obtained from the BC increases a higher parasite burden is also obtained.

## 4. Discussion

There is growing interest in the development of strategies that allow accurate determination of the parasite load in the blood of individuals with ChD [17]. The protocols used internationally have validated the use of GEB for receiving blood samples; it contains chaotropic agents and protein denaturants that cause lysis of the infectious agent, in addition to preserving the samples longer [38,39]. PCR assays use samples in GEB that incorporate a boiling step before DNA extraction to improve the sensitivity of procedures based on minicircle DNA amplification of *T. cruzi* [38,40]. GEB lyses the parasites present, releasing their genetic content and making it possible to detect only one parasite in a large volume of blood [23,38,40]. Boiling allows the release of DNA from the kinetoplast, which when broken by the effect of heat (“deconcatenation”) improves the yield of DNA extraction, especially when the process is not automated [38]. Some researchers prefer to use samples in nonboiled GEB to avoid cross-contamination between samples, but the parasite loads obtained have turned out to be lower compared to samples in boiled GEB, determined by qPCR assays [23]. The BC fraction has been used mainly to concentrate *T. cruzi* in direct microscopic studies in humans, such as in the diagnosis of congenital cases [41] or in the study of hemoparasites in animals, since it concentrates more parasite DNA than any other type of biological sample [42]. Satisfactory results have been obtained detecting *T. cruzi* using this alternative method from fresh EDTA blood samples, allowing parasites to be concentrated in this fraction, thus increasing the sensitivity of qPCR compared to whole blood samples in GEB [40,43,44].

The concentration of total DNA present in samples from BC was significantly higher in this study (*p* < 0.001) compared to the other two types of samples. However, it must be considered that this high concentration is not only increased by the DNA of circulating *T. cruzi* trypomastigotes, but also by the human genomic DNA contained in the leukocytes, where oscillating amounts of DNA must be considered over a wide range. There is considerable variability imparted by the human factor, such as the differences in the number of leukocytes of each individual, probably influenced by their state of health or immunity, among other elements [45].

BC DNA concentrations were also highly variable in our study, ranging from 24.11 ng/µL to 786.07 ng/µL, with much higher SDs than whole blood samples in boiled and nonboiled GEB. Blood samples in boiled and nonboiled GEB produced much lower concentrations than in BC, despite being obtained from the same patient. This can be explained because the whole blood samples were mixed in a 1:1 ratio with the GEB, which generates a half dilution of the sample, thus decreasing the final concentration of DNA that could be present in the sample. There were no significant differences in the concentrations present in boiled GEB and nonboiled GEB (*p* = 0.044), so we consider that the boiling step for these samples would not change the final concentration of DNA compared to if they were not boiled.

The measurement of DNA at 260/280 and 260/230 nm is a widely used tool to evaluate the correct extraction of DNA, which must safeguard the integrity of the DNA, with the least possible contamination, to obtain good performance and functionality of the analysis [46]. The A260/280 ratio makes it possible to verify contamination by proteins, which absorb at 280 nm, while at 230 nm contaminants such as chaotropic salts, phenols and carbohydrates absorb. It was determined in this study that with the A260/A280 ration the samples from BC did not present contaminants of any kind (proteins or RNA), while the contamination of samples in boiled and nonboiled GEB was mainly due to RNA. The purity obtained at A260/230 indicated contamination in all three types of samples. Phenol was ruled out as a possible contaminant, because both the samples collected, and the extraction kit used do not use this compound. However, it is possible that residual amounts of both guanidine and EDTA (components of the buffer) have remained in the samples in boiled and nonboiled GEB and are the contaminants present. Furthermore, guanidine hydrochloride is a chaotropic agent, whereas in BC samples these contaminants could only be attributed to residual amounts of EDTA (collection tube anticoagulant). The extraction kit used in this study includes a step where proteinase K is used at the beginning of the lysis of the sample. This enzyme digests proteins present in the sample, therefore obtaining DNA with a low protein content and reducing associated contaminants, as seen in all types of samples. Although the indices of purity are important indicators of the quality of the sample, the best indicator is the functionality of the recovery and purification of interest [26], which was shown in the same way when determining the presence of *T. cruzi* and its quantification, from the samples containing contaminants. Although contamination is observed in all three types of samples, when evaluating contaminant parameters, such as RNA and high contamination (which could affect DNA functionality), the BC may be the sample of choice, as it presents higher concentrations of DNA and fewer contaminants.

Sensitivity of 41% and parasite loads between 0.8 and 2 par-eq/mL have been reported for chronic ChD using qPCR-TaqMan^®^ [32], and sensitivity of 71.3%, with loads of 1.93, 2.31 and 0.1 par-eq/mL, applying qPCR-TaqMan^®^ in Argentine, Colombian, and Brazilian patients, respectively, have been reported for chronic Chagas heart disease [20]. Other authors [21], after applying qPCR-Taqman^®^ in 400 patients with chronic ChD with and without heart disease, reported 73% sensitivity in the detection of *T. cruzi,* with parasitemia ranges between 0.006 and 7.9 par-eq/mL. Our detection results are similar to other studies [33], using the same qPCR detection system, since the overall positivity of our detection by qPCR-TaqMan^®^ was 49% (26 of 53 cases), considering one, two or all three types of samples analyzed. There was greater detection analyzing the three types of samples together (38.4%); analyzing one or two sample types, the detection percentage was 30.8%. The highest and lowest detectable parasite loads were in the ranges of ≥10–100 and ≥0.01–0.09 par-eq/mL, with 3.7% and 26% of cases, respectively (Table 5). Parasitemia in the 26 cases detected ranged from very low loads (even less than 0.1 par-eq/mL), mostly in samples in nonboiled GEB, to the highest parasite loads obtained in the samples from the BC. The parasite load averages in decreasing order were BC (2.90 par-eq/mL), nonboiled GEB (2.11 par-eq/mL) and boiled GEB (0.27 par-eq/mL), with the highest SD in the BC samples. The 10 cases where circulating parasite load was detected simultaneously in the three types of samples (obtained from the same puncture per patient) presented parasitemia ranges between 0.08 and 1.29 par-eq/mL in boiled GEB, between 0.37 and 7.55 par-eq/mL in nonboiled GEB, and between 0.03 and 18.43 par-eq/mL in BC. A statistically significant difference was only found between the means of parasitemia obtained in boiled GEB (0.45 par-eq/mL) and nonboiled GEB (2.42 par-eq/mL) (*p* = 0.013). Despite the higher average loads found in BC (4.85 par-eq/mL), there were no statistically significant differences with the other groups, due to the high intragroup variability (Figure 3).

In the seven cases where the parasite load was quantifiable (≥0.1 par-eq/mL) in the three types of samples, the averages were 0.58 par-eq/mL in boiled GEB, 3.22 par-eq/mL in nonboiled GEB and 6.32 par-eq/mL in BC, without significant differences, described in the results. Others studies of validation of qPCR-Taqman^®^, used nonboiled GEB and boiled GEB blood samples from 63 Bolivian patients and 63 Argentine patients with chronic ChD, detecting 60.3% positivity in nonboiled GEB samples and 76.5% in boiled GEB, with lower parasite loads in nonboiled GEB samples, where only three of 38 positive samples were quantifiable (1.25, 1.44 and 1.45 log_10_ par-eq/10 mL), indicating that most of the patients in this group presented very low parasite loads. Many of the patients gave detectable but not quantifiable results according to their method [23]. They estimated that the incubation of the samples for 15 min favored the fragmentation or deconcatenation of the minicircles and the distribution of individual minicircles throughout the sample volume, allowing the processing of small volumes (100 µL) with satisfactory sensitivity, thus obtaining a slightly higher sensitivity using boiled GEB versus nonboiled GEB (0.70 versus 0.46 par-eq/mL, respectively; *p* = 0.044). They suggested that the higher parasite loads found in these cases were partially influenced by the boiling step. However, they considered that the heating procedure could increase the risk of cross-contamination between samples, leading to false positives, so they continued their qPCR validation using nonboiled spiked samples [25]. This study differs from ours, since we detected lower loads in boiled GEB, obtaining intervals between 0.04 and 0.08 par-eq/mL in 10 of the 19 detected cases, that is, detectable but not quantifiable according to our study method, while in nonboiled GEB all samples presented parasite loads above 0.1 par-eq/mL. Using mostly EDTA blood samples, in addition to whole blood, to determine the effect of BC concentration on the sensitivity of the qPCR-Taqman^®^ assay in patients with suspected acute or reactivated ChD living in the USA, found detectable levels of *T. cruzi* DNA in both whole blood and BC in 26 samples (from 10 patients) [40]. They reported relatively higher concentrations in BC, which is consistent with our study, despite the fact that we used samples from patients with chronic ChD where parasitemia is low and fluctuating. On the other hand, another nine samples (from six patients) were positive only for BC, therefore, 26% of the positive samples would have been reported as negative for *T. cruzi* had the BC not been tested [40]. These observations are consistent with our results, since three of the 26 cases we detected would have gone undetected if BC had not been analyzed.

Finally, studies of patients with chronic ChD using whole blood samples in boiled GEB for SatDNA and qPCR assay, reported parasitemia between 1.61 and 2.46 par-eq/mL, while in blood culture they obtained loads between 1.68 and 15.54 par-eq/mL [17]. In our study, the intervals of parasitemia obtained in the samples in boiled GEB and nonboiled GEB are similar to these previous studies, while the parasitemia obtained from BC is similar to the loads obtained in blood cultures.

We found a certain association between the total DNA concentration and the parasite load in all types of samples considering the seven quantifiable positive cases, being higher for BC samples. Similarly to this study, other investigators have estimated that, based on DNA extracted from BC in a blood sample containing *T. cruzi* trypomastigotes, BC should concentrate more parasite DNA than the corresponding volume of whole blood [40].

This study determined that samples from BC present higher concentrations of both total DNA and parasite load. Samples in nonboiled GEB had higher parasite load averages than those in boiled GEB. We note that parasite loads of less than 0.1 par/eq/mL were probably not detected in the nonboiled samples because they were not boiled and thus avoided DNA damage from being subjecting to heat, as in the BC that was not boiled either. Despite this, we cannot reliably determine this difference because there were no statistically significant differences between the average parasitemia in the groups in the seven quantifiable cases, so we consider it necessary to increase the number of samples that meet this criterion (detectable in the three types of samples) in subsequent studies. However, there are intrinsic characteristics in the samples from these patients that could influence the detection and quantification of the parasite load by qPCR, such as the genetic history of the host and their immunological status, which may play a role in the control of parasite replication, and also a factor related to the DTU of *T. cruzi* circulating in the patient [23,47]. The variables mentioned that were not addressed in the present study could have influenced the levels of parasite load obtained in the different types of samples.

Finally, due to the low parasite loads obtained in this type of patient, it is necessary to continue implementing more precise methodologies. One alternative could be the use of digital PCR, a new technology of high resolution and absolute quantification, which would allow comparison of detection limits with qPCR, especially when it is necessary to assess the parasite load after patient treatment. In addition, it could be very useful to take serial samples from patients, at different periods of time, to evaluate fluctuations in parasitemia, thus increasing sensitivity.

## 5. Conclusions

The application of qPCR-Taqman^®^ in blood samples processed under different protocols for obtaining and processing samples confirms the low circulating parasitemia in individuals with chronic ChD, despite the improvement in concentration conditions. It is concluded that the samples obtained in GEB and later boiled have the disadvantage of presenting impurities and low concentrations of DNA, while the BC samples have higher purity and DNA concentration. Given that there were no statistically significant differences in the determination of parasite load between the three types of samples analyzed, it is proposed to consider all the advantages that BC has as a biological sample to detect circulating *Trypanosoma cruzi* by PCR or qPCR. While there is no biological marker of parasitemia for Chagas disease, the challenge is to improve the parasitological techniques available today. For example, to treat the BC with guanidine before DNA extraction to increase the sensitivity.

## Figures and Tables

**Table 1:** Sequences and primers used to detect *Trypanosoma cruzi*.

Primer	Sequence 5′---->3′
Cruzi 1	ASTCGGCTGATCGTTTTCGA
Cruzi 2	AATTCCTCCAAGCAGCGGATA
Cruzi 3	FAM-CACACACTGGACACCAA-NFQ-MFB

[18,19,23,32].

**Table 2:** DNA purity at 260/280 nm and 260/230, depending on sample type.

DNA Purity 260/280 and 260/230	Boiled GEB Samples 260/280–260/230		Nonboiled GEB Samples 260/280–260/230		BC Samples 260/280–260/230	
Optimum purity	42	35	38	35	51	30
Acceptable purity	1	7	9	5	2	13
Contaminated with aromatic compounds (proteins)/Contaminated with chaotropic salts or carbohydrates	0	7	0	6	0	10
Probable RNA contamination/ Highly contaminated with chaotropic salts or carbohydrates	10	4	6	7	0	0

Ranges of reference (www.bancoadn.org 29 November 2023 University of Salamanca, 2020): Optimum DNA purity 260/280: Index ≥ 1.8–2.1, 260/230: Index > 2–2.2; Acceptable DNA purity: 260/280: Index ≥ 1.6–1.7, 260/230: Index > 1.8; DNA contaminated with aromatic compounds (proteins): 260/280: Index < 1.6, DNA contaminated with chaotropic salts or carbohydrates: 260/230: Index < 1.8; RNA contamination: 260/280: Index > 2.1; 260/230: DNA highly contaminated with chaotropic salts or carbohydrates: Index < 1.5.

**Table 3:** Percentage of detection of *Trypanosoma cruzi* DNA according to sample type in a total of 53 patients with chronic Chagas disease.

Samples n	Boiled GEB Positive/Negative	Non-Boiled GEB Positive/Negative	BC Positive/Negative	Detection %
10	Positive	Positive	Positive	18.86
3	Positive	Positive	Negative	5.66
2	Positive	Negative	Positive	3.77
3	Negative	Positive	Positive	5.66
4	Positive	Negative	Negative	7.54
1	Negative	Positive	Negative	1.88
3	Negative	Negative	Positive	5.66
27	Negative	Negative	Negative	50.90
53	Positives = 19 35.8%	Positives = 17 32%	Positives = 18 34%	100%

**Table 4:** Quantification of parasite load of *Trypanosoma cruzi* by qPCR-Taqman^®^ in three types of samples of 26 patients with chronic Chagas disease.

Patient Information			Parasite Load (par-eq/mL)		
Sample Number	Gender	Age	Boiled GEB Samples	Nonboiled GEB Samples	BC Samples
1	F	56	0.62	1.18	18.43
2	M	83	1.15	7.55	9.00
3	F	78	0.15	2.59	1.19
4	M	58	0.08	0.39	0.86
5	M	58	0.42	4.72	2.09
6	F	57	1.29	3.49	12.17
7	M	75	0.25	0.65	0.43
8	F	83	0.21	2.36	0.94
9	F	72	0.08	0.37	3.40
10	F	59	0.25	0.91	0.03
11	F	56	0.08	3.93	ND
12	F	68	0.09	2.72	ND
13	M	68	0.06	0.51	ND
14	F	55	0.11	ND	1.37
15	M	69	0.08	ND	0.64
16	F	60	ND	0.76	0.80
17	F	63	ND	0.83	0.38
18	F	62	ND	0.52	0.20
19	F	72	0.05	ND	ND
20	F	36	0.09	ND	ND
21	F	49	0.06	ND	ND
22	M	54	0.04	ND	ND
23	F	66	ND	2.37	ND
24	M	86	ND	ND	0.09
25	F	62	ND	ND	0.08
26	M	56	ND	ND	0.04

ND: not detectable.

**Table 5:** Parasitemia ranges according to sample type.

Range (par-eq/mL)	Boiled GEB Samples	Nonboiled GEB Samples	BC Samples
≥10–100	0	0	2
≥1–9	2	9	5
≥0.1–0.99	7	8	7
≥0.01–0.09	10	0	4
Total samples	19	17	18

## Data Availability

The data presented in this study are contained in this article.
